# Complete mitochondrial genome of *Promethis valgipes valgipes* (Marseul) (Insecta: Coleoptera: Tenebrionidae)

**DOI:** 10.1080/23802359.2020.1861564

**Published:** 2021-02-11

**Authors:** Yu Bai, Jun Chen, Guoyong Li, Jianlin Luo, Hui Wang, Yang Yang, Sheng Liang, Bocheng Ouyang

**Affiliations:** a College of Mathematics & Information Science, Guiyang University, Guiyang, China; b School of Electronic & Communication Engineering, Guiyang University, Guiyang, China; c Guizhou Provincial Key Laboratory for Rare Animal & Economic Insects of the Mountainous Region, Guiyang University, Guiyang, China

**Keywords:** *Promethis valgipes valgipes*, Tenebrionidae, mitochondrial genome, fungus-eating insect, mitogenome

## Abstract

*Promethis valgipes valgipes* (Marseul) is one of the important fungus-eating beetle distributed in central China, Korea, and Japan. Beetles were obtained from Pizhou City and the species’ mitochondrial genome was characterized (GenBank accession number MW201671). The mitogenome consists of a circular DNA molecule of 15,801 bp, with 68.51% AT content. It comprises 13 protein-coding genes (PCGs), 22 tRNA genes, and two rRNA genes. The PCGs have typical ATN (Met) start codons, except *nad1* (TTG as start codon), and are terminated by typical TAN stop codons.

*Promethis valgipes valgipes* (Marseul) is an important fungus-eating beetle that is distributed in central China, Korea, and Japan (Hua et al. 2006). Its mitochondrial genome has not previously been sequenced completely. We herein characterize the first complete mitogenome of *P. valgipes valgipes* with the aim of illuminating its molecular evolution and taxonomic affinities.

Specimens of adult *P. valgipes valgipes* were collected in Paoche town (118.04°N, 34.32°E), Pizhou City, Jiangsu Province, China, on 21 July 2019, and deposited by the first author in the animal specimen room of Guiyang University with specimen accession number: GYU-20190721-001. Genomic DNA was isolated and fragmented to build a genomic library with an insert size of 400 bp that was sequenced (paired-end, 2 × 150 bp) using an Illumina HiSeq 4000 (Illumina, Inc., San Diego, CA). We obtained approximately 3895.37 Mb of raw data, of which 3652.81 Mb (93.77%) were high-quality, clean data. The genome was assembled *de novo* using A5-miseq v20150522 (https://github.com/koadman/docker-A5-miseq) (Coil et al. 2015) and SPAdes v3.9.0 (http://cab.spbu.ru/software/spades/) (Bankevich et al. 2012).

The mitogenome of *P. valgipes valgipes* (GenBank accession number: MW201671) consists of a circular DNA molecule of 15,801 bp (27.95% A, 40.56% T, 11.75% C, and 19.74% G; 68.51% AT content). Using Perna and Kocher’s formula (Perna and Kocher 1995), the AT- and GC-skews of the major strands of the mitogenome were calculated to be approximately 0.184 and −0.254, respectively. The AT-rich region in the mitogenome is of 1213 bp, with a 78.40% AT content, and is located between the genes encoding srRNA and tRNA-Ile.

The mitogenome of *P. valgipes valgipes* contains 13 protein-coding genes (PCGs), 22 tRNA genes, and two rRNA genes, which were annotated using the MITOS web server (http://mitos.bioinf.uni-leipzig.de/) (Bernt et al. 2013). The order and orientation of the functional areas are the same as that in the mitogenomes of *Tenebrio obscurus* (Bai et al. 2018), *Zophobas atratus* (Bai et al. 2019), and *Blaps rhynchoptera* (Yang et al. 2019). All 13 PCGs have typical ATN (Met) start codons, except *nad1*, which has a TTG start codon. Five genes (*nad3*, *atp8*, *cox2*, *cox1*, and *nad2*) have an ATA start codon; one gene (*nad6*) has an ATC start codon; five genes (*cob*, *nad4l*, *nad4*, *cox3*, and *atp6*) have an ATG start codon; and one gene (*nad5*) has an ATT start codon. All 13 PCGs have typical TAN stop codons. Nine genes (*cob*, *nad4l*, *nad5*, *cox3*, *atp8*, *cox1*, *atp6*, *nad2*, and *nad6*) have a TAA stop codon; two genes (*nad1* and *nad3*) have a TAG stop codon; and two genes (*nad4* and *cox2*) have an incomplete stop codon consisting of a T that is completed by the addition of A nucleotides to the 3′ end of the encoded mRNA. The 22 genes encoding tRNAs are interspersed throughout the coding region and range from 59 bp (tRNA-Ser) to 70 bp (tRNA-Lys). The genes encoding lrRNAs and srRNAs are 1261- and 760-bp long, respectively.

To validate the phylogenetic position of *P. valgipes valgipes*, its mitochondrial PCGs and those of 12 other species of Tenebrionidae were used to construct a phylogenetic tree with the MEGA X software (Kumar et al. 2016) via the maximum-likelihood method (Figure 1). Our study provides fresh insights into the mitogenome of *P. valgipes valgipes*, providing essential genetic and molecular data for further phylogenetic and evolutionary analysis of the Tenebrionidae.

**Figure 1.**
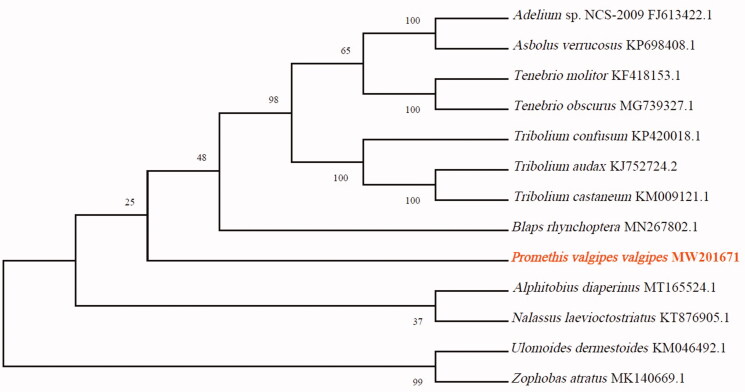
Maximum-likelihood phylogenetic tree of *Promethis valgipes valgipes* and 12 other Tenebrionidae beetles based on the protein-coding regions of their mitogenomes.

## Data Availability

Mitogenome data supporting this study are openly available in GenBank at: https://www.ncbi.nlm.nih.gov/nuccore/MW201671. Associated BioProject, SRA, and BioSample accession numbers are https://www.ncbi.nlm.nih.gov/bioproject/673297, https://www.ncbi.nlm.nih.gov/sra/SRX9403177, and https://www.ncbi.nlm.nih.gov/biosample/SAMN16598392, respectively.
